# Nasal Chondromesenchymal Hamartoma with Skull Base and Orbital Involvement: Case Presentation

**DOI:** 10.7759/cureus.2892

**Published:** 2018-06-28

**Authors:** Denis A Golbin, Anastasia P Ektova, Maxim O Demin, Nikolay Lasunin, Vasily A Cherekaev

**Affiliations:** 1 Skull Base and Craniofacial Surgery, N.N. Burdenko National Medical Research Center for Neurosurgery, Moscow, RUS; 2 Pathology, Russian Children's Clinical Hospital, Moscow, RUS; 3 Pediatric Neurosurgery, N.N. Burdenko, Moscow, RUS; 4 Neuro-Oncology, N.N. Burdenko National Medical Research Center for Neurosurgery, Moscow, RUS

**Keywords:** nasal chondromesenchymal hamartoma, skull base tumor, pediatric tumors, endoscopic endonasal approach, immunohistochemistry

## Abstract

Nasal chondromesenchymal hamartoma (NCMH) is a rare benign tumor of the sinonasal tract in children with possible orbit and skull base involvement. We present the 57th published observation of this kind of tumor. A 25-month-old female patient presented with recurrent mass lesion of the sinonasal tract. According to her history, she had feeding difficulties and nasal obstruction since birth. She underwent partial resection at eight months of age via transfacial approach in the local hospital. Due to progression of tumor remnants, a second surgery was performed using an endoscopic endonasal approach resulting in subtotal resection. At 12 months of follow-up, a good postoperative result was observed with no signs of tumor progression despite incomplete resection. Histological and immunohistochemical examination of the biopsy specimens is presented. Comparison of specimens obtained from each of the two surgeries showed a difference in histological patterns. Endoscopic endonasal approach is the mainstay of surgical management. In case of incomplete resection, careful follow-up MRI studies should be recommended.

## Introduction

Nasal chondromesenchymal hamartoma (NCMH) is a rare benign tumor of the sinonasal tract in children with possible orbit and skull base involvement. Morphologically this tumor represents a heterogeneous mixture of spindle cells, collagen fibers, and irregular islands of osseous and chondroid tissue [1].

After review of the literature we found 56 published cases of NCMH in single reports and small series. Majority of cases occurred in infants and young children, much rarely in elder people. The most common symptoms include breast-feeding difficulty and nasal obstruction, and clinical presentation is non-specific [2]. Since the vast majority of all NCMH cases are diagnosed in young age, the lesion is considered to be congenital [3].

Complete excision is treatment of choice, and good results are usually achieved. NCMH is amenable for endoscopic resection [4]. Incomplete excision could result in tumor recurrence, however only few studies reported NCMH relapse [2, 5-6]. We present a case of NCMH in a child treated surgically twice due to incomplete primary resecion; a morphological analysis is included.

## Case presentation

**Figure 1:**
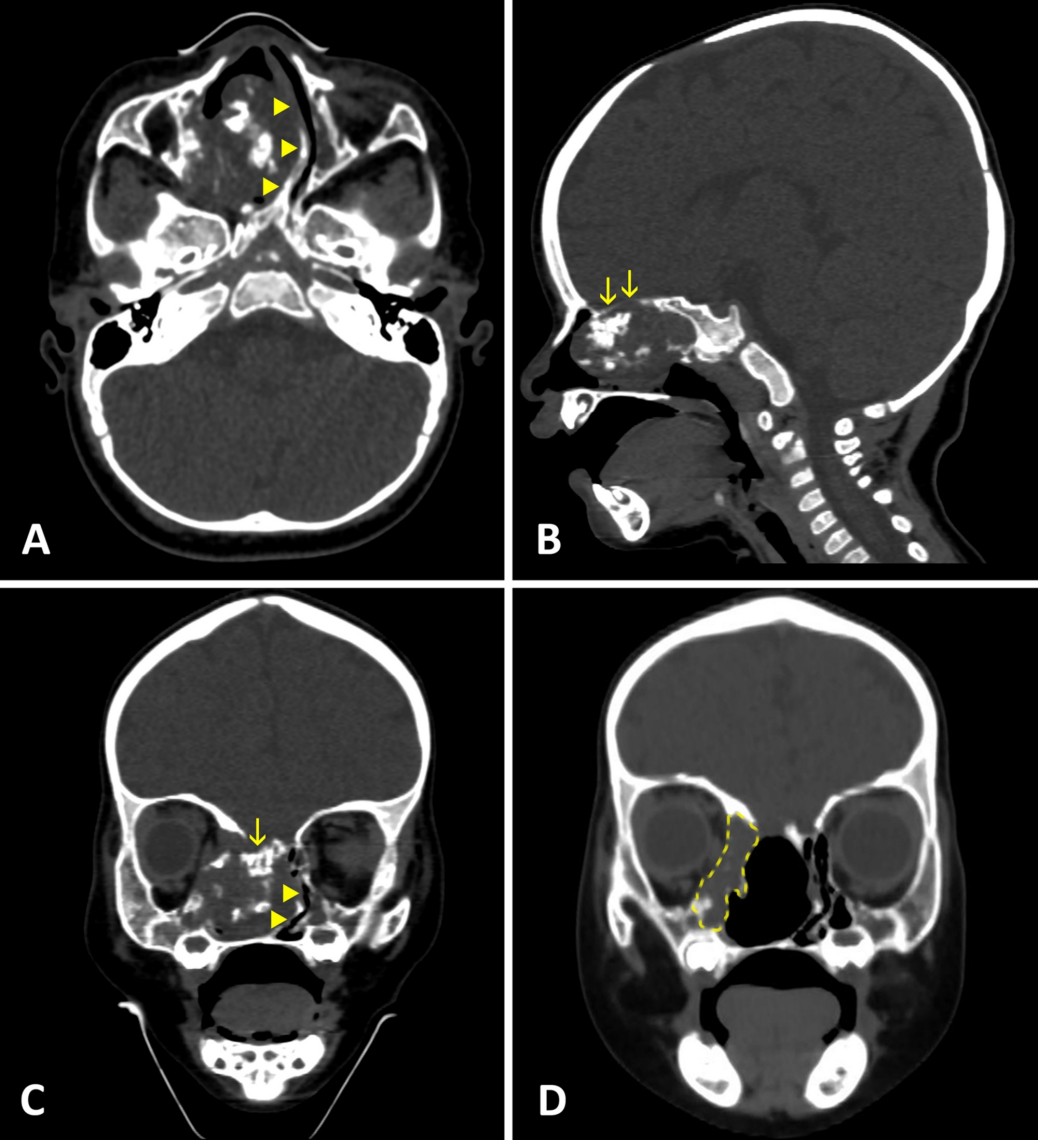
CT scans before (A-C) and after (D) the first surgery The lesion occupies the right nasal cavity, ethmoid labyrinth, maxilla with limited orbital, and skull base extension (arrows). Calcifications are visible within the tumor mass. Displaced nasal septum is marked by arrowheads. Postoperative coronal CT scan (D) shows unresected lateral portion of the tumor.

A 25-month-old female patient presented with recurrent mass lesion of the sinonasal tract. According to her history, she had feeding difficulties and nasal obstruction since birth. Microphthalmia on the right side was also noticed. Examination revealed mass lesion in the right nasal cavity and maxilla, however, biopsy was noninformative. Then, in October 2015 at the age of eight months, the patient was admitted to the department of maxillofacial surgery of local pediatric hospital. Computed tomography (CT) scans were obtained demonstrating a widespread tumor in the right nasal cavity with severe dislocation of the nasal septum, involving the right maxilla, ethmoid labyrinth, orbit, and cranial base (Figure 1A, 1B, 1C). In November 2015, the lesion was resected via a lateral rhinotomy in a piecemeal fashion until the bony boundaries of the maxillary antrum were reached around the tumor mass. Postoperative CT scans showed tumor remnants along the lateral nasal wall in proximity to the orbit (Figure 1D). No complications occurred after surgery. Histologic examination diagnosed chondromesenchymal hamartoma.

**Figure 2:**
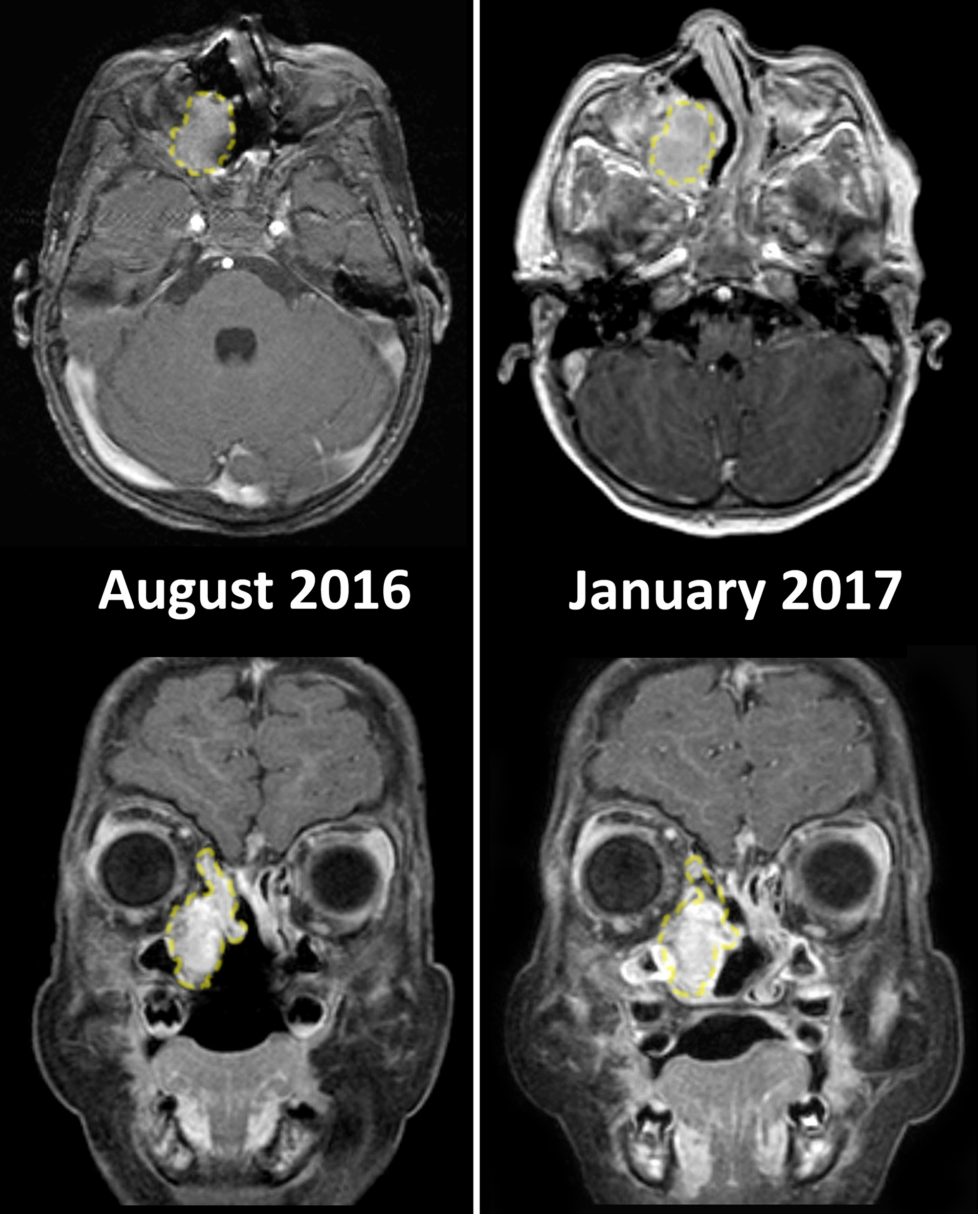
Follow-up MRI after the first surgery In August 2016, (left) images revealed recurrence of the tumor eight months after surgery, however, without significant mass effect. Comparison of the corresponding scans between August 2016 and January 2017 (right) suggests the increase in dimensions of the lesion, which meant that it was not stable. To demonstrate the difference in size, the outline of the tumor on the earlier scans is positioned over the later images (dashed line).

The patient presented at N.N. Burdenko National Research Center for Neurosurgery (Moscow, Russia) to obtain consultations concerning the management of the remaining lesion. New MRI obtained in August 2016 (Figure 2, left) showed remnants of the tumor without any deficit, and further follow-up was recommended.

The histological specimens were examined in the pathology department, and the diagnosis of NCMH was confirmed.

**Figure 3:**
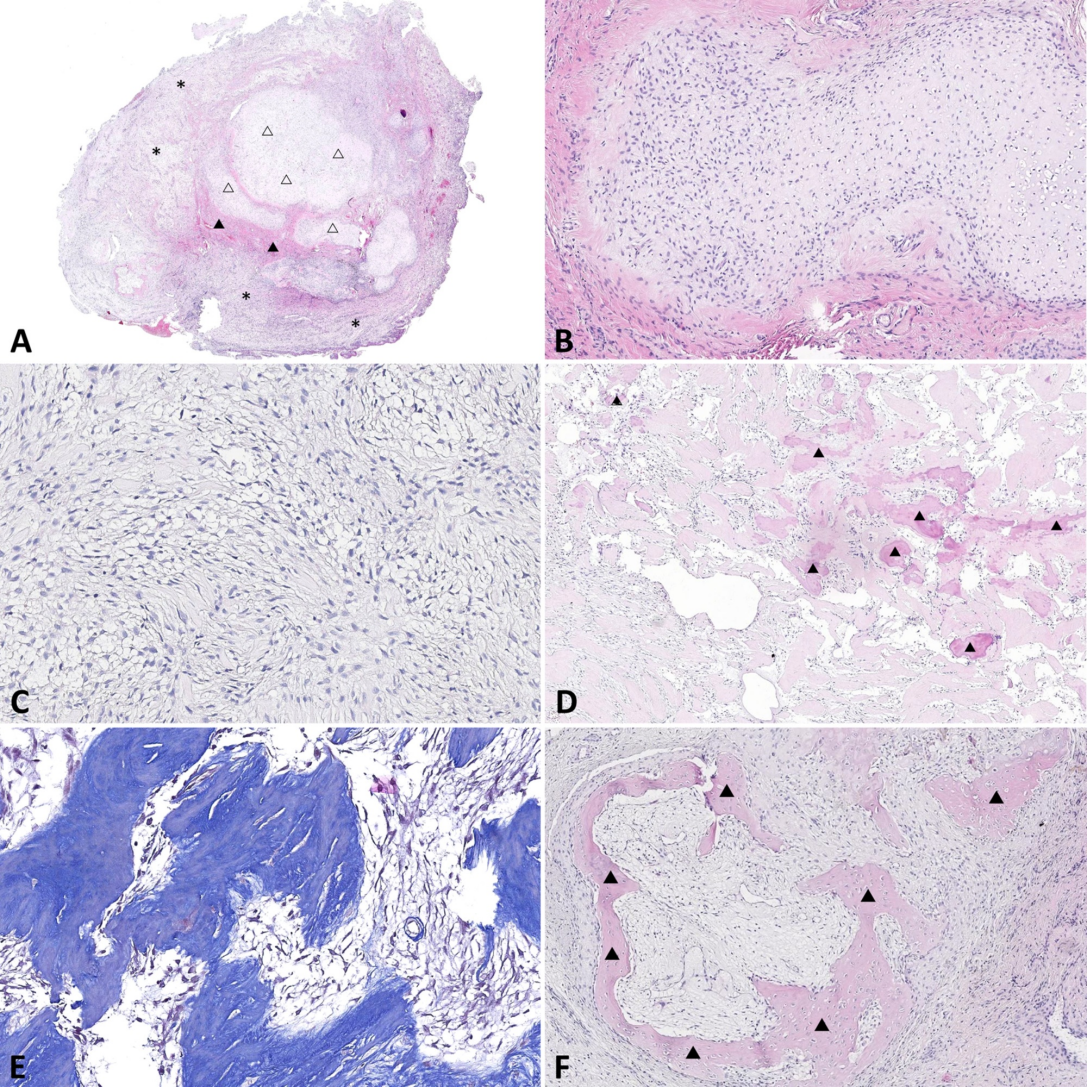
Pathology of the lesion obtained from the first surgery A: H&E staining, x 10. The lesion contains chondroid (void triangles), osseous (solid triangles), and mesenchymal (asterisk) components. B: H & E staining, x 200. Cartilaginous focus is visible in the center of the image. C: H & E staining, x 400. Mesenchymal component at high magnification. D: H & E staining, x100. Bony trabeculae are indicated by solid triangles. E: Masson’s trichrome staining for demonstration of the osteoid matrix, x 400. F: H & E staining, x 100: pre-existing bone tissue is highlighted by solid triangles.

On low magnification, the resected material showed different histological patterns. It consisted of cellular cartilaginous islands and areas that contained fibro-osseous and mesenchymal components (Figure 3A). The cartilaginous component was composed of cellular cartilage foci with a hyaline cartilaginous matrix. The cells of that foci had a very low level of mitotic activity, and no signs of atypia were found (Figure 3B).

A mesenchymal component was represented by quite cellular zones consisting of plump fibroblast-like cells without any mitotic figures and atypia (Figure 3C). Multiple bone trabeculae (Figure 3D) and irregular osteoid matrix (blue on Masson – Figure 3E) were found upon the connective tissue background. Areas of preexisting bone tissue with lamellar structure were present (Figure 3F).

**Figure 4:**
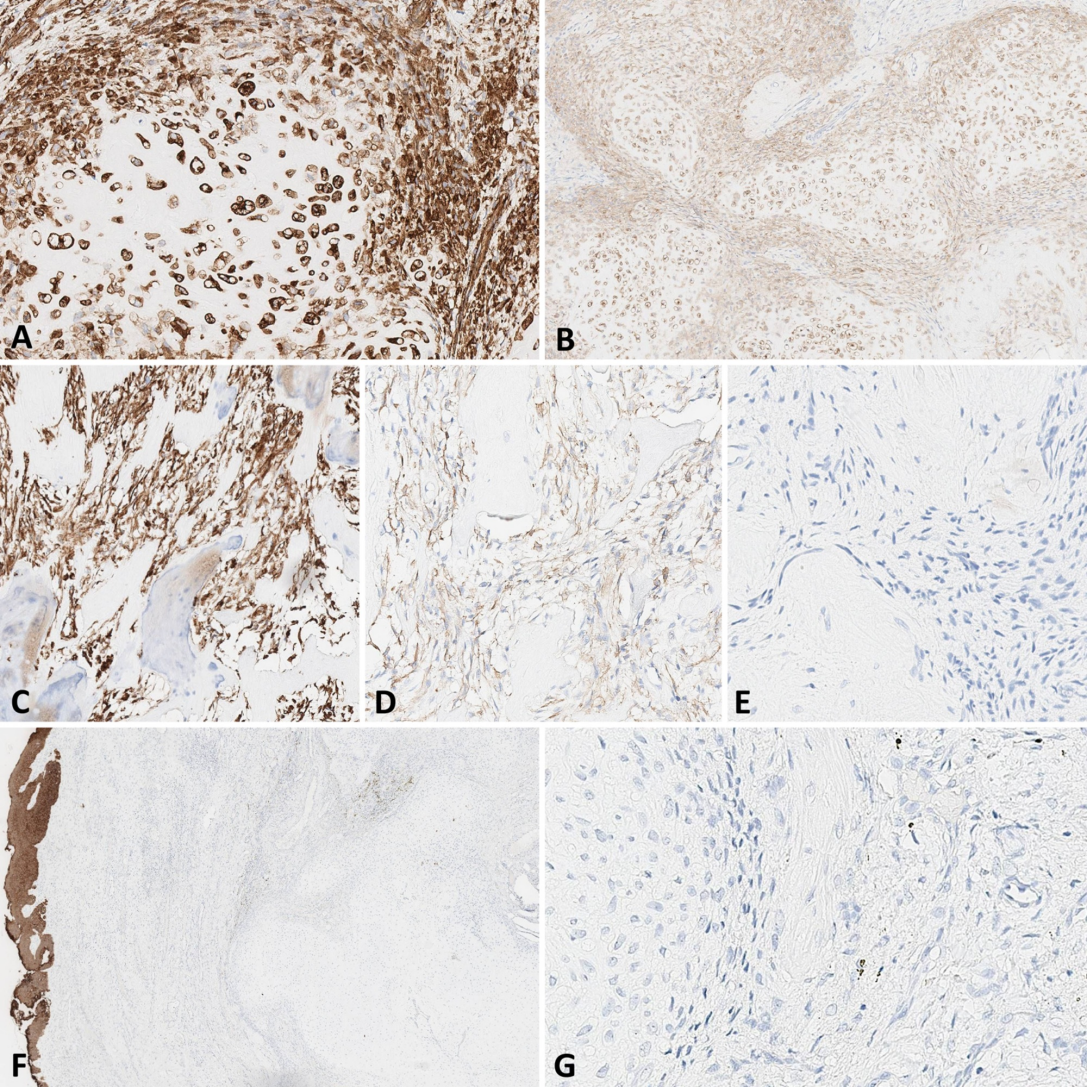
Immunohistochemical examination of the specimen A: expression of vimentin in the cartilaginous component, x 400. B: expression of S-100 protein in the cartilaginous component, x 100. C: expression of vimentin in the mesenchymal component, x 200. D: expression of SMA in the mesenchymal component, x200. E: absence of MDM2 expression, x400. F: presence of cytokeratin expression in the mucosal epithelium (brown), x10. G: low proliferative activity resulting in lack of Ki-67 expression, x400

Immunohistochemical study with antibodies to SMA, S-100 protein, vimentin, MDM2, CDK4, desmin, CD34, and Ki-67 was performed. The cells of the cartilaginous component were strongly positive for vimentin (Figure 4A) and S-100 protein (Figure 4B). The cells of the mesenchymal component are positive for vimentin and focally positive for SMA (Figures 4C and 4D). Immunohistochemical reactions with MDM2 (Figure 3E) and PanCK (AE1/AE3) were negative, positive control for PanCK was present in the mucosal epithelium (Figure 4F). Proliferative activity was very low according to Ki67 expression (Figure 4G).

In January 2017 (Figure 2, right) the next follow-up MRI study showed a progression of the lesion, and surgical treatment was indicated. Since the tumor was limited to the nasal cavity, ethmoid labyrinth, maxillary sinus, and was situated extradurally, the second surgery was performed using endoscopic endonasal technique (March 2017). No distinct margins of the lesion were detectable intraoperatively, therefore the superior portion of the tumor was left in order to avoid skull base penetration. Post-operative course was uneventful, no cerebrospinal fluid leak was detected.

**Figure 5:**
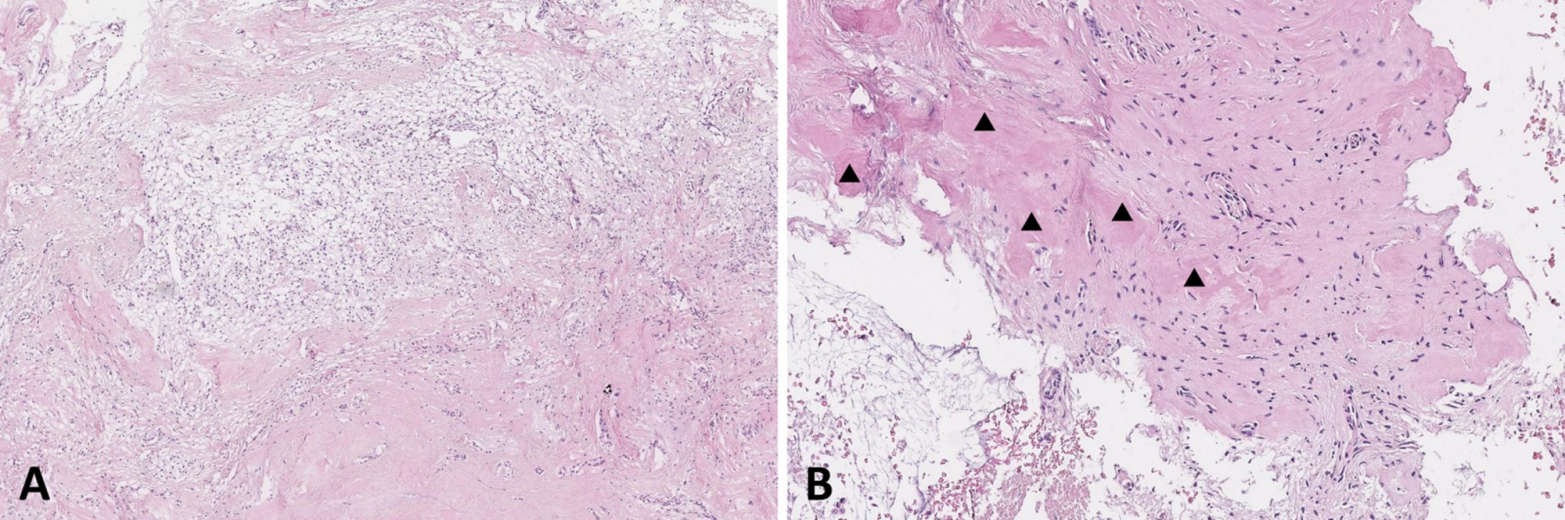
Pathology of the recurrent lesion obtained from the second surgery A: H & E staining, x100. On the background of connective tissue (pinkish areas) reactive inflammatory infiltration is present. B: H & E staining, x200. Osteoid matrix is indicated by solid triangles

The material from the second surgery was represented by hypocellular connective tissue with reactive inflammatory infiltration (Figure 5A), and the same irregular osteoid matrix (Figure 5B). In this case, the cartilaginous component was not found.

**Figure 6:**
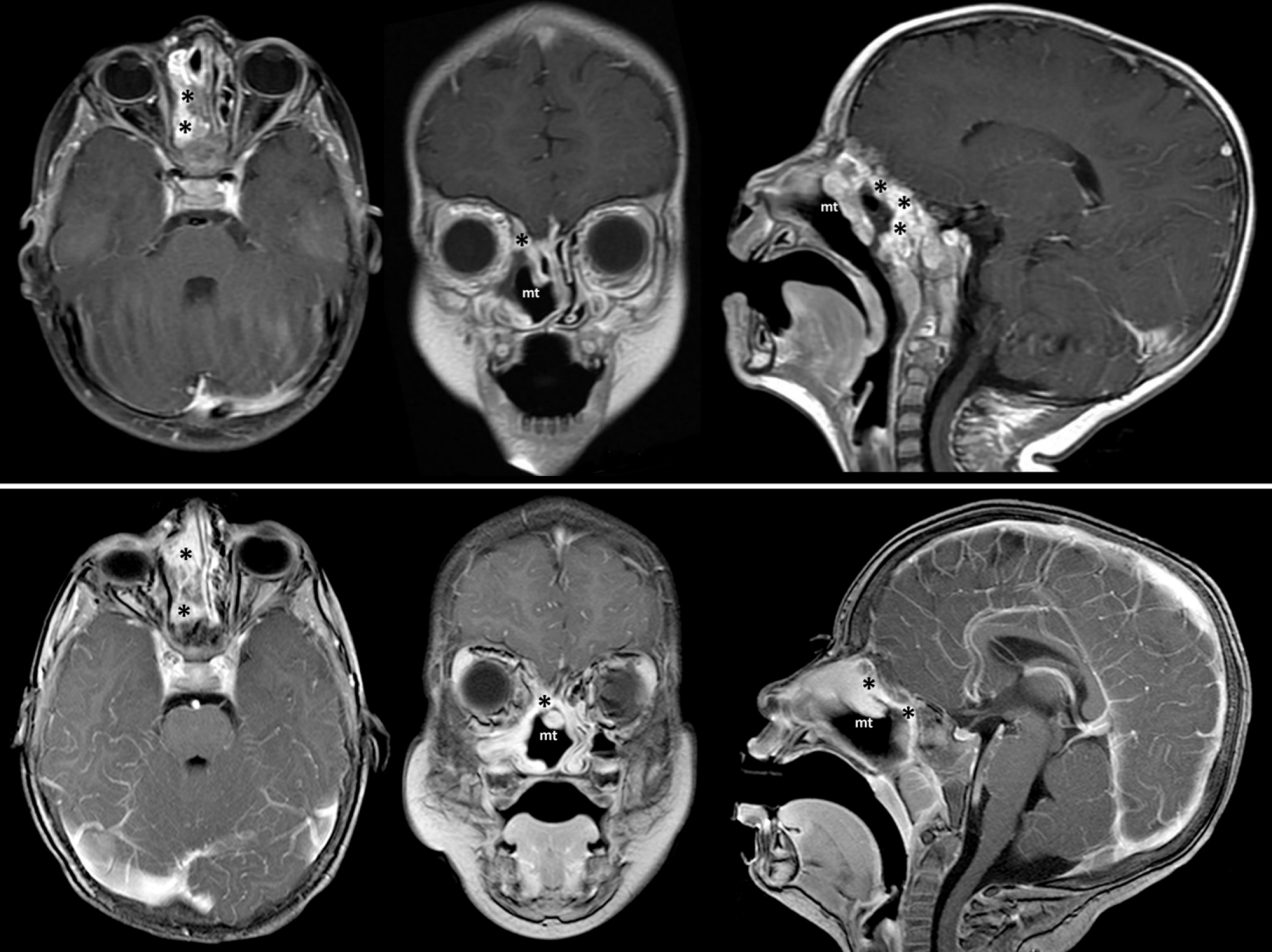
Follow-up MRI. Top: at six months of follow-up after the second surgery. Bottom: at 12 months after the second surgery Lateral nasal wall is completely free of tumor mass; however, the remnants are visible along the ethmoid roof and in the sphenoid sinus (asterisks). There is no evidence of tumor progression. Decrease of inflammatory changes is observed. (mt – middle turbinate)

At six months of follow-up, the magnetic resonance imaging (MRI) demonstrated clear nasal cavity and remaining tumor mass in the ethmoid roof and sphenoid sinus (Figure 6, top). At one year, after the second surgery inflammatory changes had regressed, the tumor is stable and exhibits no increase in the volume (Figure 6, bottom).

## Discussion

NCMH was described as a distinct lesion back in 1998 by McDermott MB et al. [3] and has also been called “chondroid hamartoma”, “mesenchymoma”, “nasal hamartoma”, and other names in the literature. His study represented a group of children diagnosed with rare and benign tumors involving sinonasal tract as well as craniofacial structures (cribriform plate, cranial base dura). Common symptoms in his series included breast-feeding difficulty and nasal obstruction. According to histological reports, tumor masses were presented as heterogeneous mixture of spindle cells, collagen fibers, and irregular islands of osseous and chondroid tissue. NCMH generally is diagnosed in the newborn period and is much rarer in adolescents [7]. Malignant metamorphosis of tumor is uncommon [8].

To date, a total of 56 cases are reported in literature in single reports and small series. NCMH is generally diagnosed in the newborn period and far more rarely in adolescents. Common symptoms correlated with localization of lesions and presented as breast-feeding difficulty, nasal obstruction, rhinorrhea, epistaxis, and middle ear effusion. Moreover, involvement of the orbit or cranial cavity may cause oculomotor disorders, proptosis or enophthalmos as well as neurologic deficit [2]. In other words, clinical appearance and symptoms are not specific nor sufficient to diagnose NCMH.

Differential diagnosis includes hemangioma, angiofibroma, antrochoanal polyp, nasoethmoidal encephalocele, or meningoencephalocele along with other rare benign pediatric mass lesions. Radiological diagnosis is based on CT and MRI. Head CT and MRI focusing on the sinonasal tract help to reveal the site of origin, true extent of the lesion, relationship with critical structures. Lesions are described as heterogeneous with low signal intensity on T1-weighted images and have high signal intensity on T2-weighted images with significantly heterogeneous contrast enhancement. CT modality shows heterogeneous soft-tissue masses with predominantly solid and cystic components [9-10]. Microscopic picture of NCMH is presented by irregular islands of mature as well as immature hyaline cartilage with occasional chondrocytes. The cartilage islands have well-demarcated borders with the surrounding stromal tissues, which have a myxoid background. No atypical mitotic figures or malignant characteristics have been seen. Spindle cells with occasional mitotic figures are reported. Almost all NCMH samples [1] demonstrated immunohistochemical positivity to SMA, S-100, and vimentin.

Because the vast majority of all patients are commonly diagnosed with NCMH in young age, the lesion is considered to be congenital. This theory is straightened by original McDermott’s series [3], which included a seven-year-child with pleuropulmonary blastoma and NCMH. In certain papers [7] authors link NCMH with chronic inflammatory process (e.g. chronic sinusitis). However, as there is no clear pathogenesis described, it is difficult to define whether NCMH is a primary or secondary event to chronic inflammatory process. Moreover, recent cytogenetic research [11] has discovered that lesion arises in association with Carney I complex and may possibly harbor PRKAR1A loss, coding 1-alpha regulatory protein kinase A subunit. Balanced-appearing translocation t(12;17)(q24.1;q21) was identified as the sole clonal karyotypic anomaly in a case of NCMH arising in an 11-year-old boy. This finding supports the hypothesis that NCMH represents a true neoplasm.

Complete excision is the treatment of choice, and good results are usually achieved. NCMH is amenable for endoscopic resection [4]. Total endoscopic resection should be considered as main treatment in cases of NCMH nowadays. Incomplete excision could result in tumor recurrence, at least it helps in restoring the nasal passage; however, there are only few cases reported on NCMH relapse [2, 5-6]. Thus, there is no need of adjuvant radiotherapy or chemotherapy. At the same time, some data suggest that complete resection cannot absolutely eliminate the risk of recurrence [12]. 

In the described case, significant portion of the tumor was left at the first surgery despite wide exposure. Remaining masses along the lateral nasal wall progressed then, and reoperation was indicated. However, incomplete primary resection hindered the possibility to accomplish safe clear-margin resection at the second surgery. Nevertheless, it turned out to be more successful, and tumor masses were left only along the skull base for the sake of risk avoidance. This experience supports the priority of endoscopic endonasal approach for surgical management of NCMH in pediatric patients of any age.

The course of the disease was consistent with the behavior of a benign tumor. In comparison with the material from the first surgery, the tissue obtained during the second resection lacked cartilaginous component. This specimen was composed mostly of mesenchymal tissue with myxoid areas and focal inflammatory changes.

## Conclusions

We presented the 57th published report of NCMH. Due to its typical behavior for benign tumors, maximally complete surgical resection is the treatment of choice. Endoscopic endonasal approach is the mainstay of surgical management. In case of incomplete resection, careful follow-up MRI studies should be recommended. We demonstrated that the histological pattern of the lesion at repeated surgeries may be different from the primary.

## Appendices

DG (Denis A. Golbin) composed the article, wrote the case presentation and prepared figures. AE (Anastasia P. Ektova) carried out the pathology studies, participated in the case presentation and preparation of figures. MD (Maxim O. Demin) wrote the discussion section. NL (Nikolay Lasunin) participated in the case presentation and discussion section. VC (Vasily A. Cherekaev) conceived of the study and helped to draft the manuscript. All authors read and approved the final manuscript.

## References

[1] Johnson C, Nagaraj U, Esguerra J, Wasdahl D, Wurzbach D (2007). Nasal chondromesenchymal hamartoma: radiographic and histopathologic analysis of a rare pediatric tumor. Pediatr Radiol.

[2] Mattos J, Early S (2017). Nasal chondromesenchymal hamartoma: a case report and literature review. Int J Pediatr Otorhinolaryngol Extra.

[3] McDermott MB, Ponder TB, Dehner LP (1998). Nasal Chondromesenchymal Hamartoma. Am J Surg Pathol.

[4] Nakaya M, Yoshihara S, Yoshitomi A, Baba S (2017). Endoscopic endonasal excision of nasal chondromesenchymal hamartoma with intracranial extension. Eur Ann Otorhinolaryngol Head Neck Dis.

[5] Kim DW, Low W, Billman G, Wickersham J, Kearns D (1999). Chondroid hamartoma presenting as a neonatal nasal mass. Int J Pediatr Otorhinolaryngol.

[6] Mason KA, Navaratnam A, Theodorakopoulou E, Chokkalingam PG (2015). Nasal chondromesenchymal hamartoma (NCMH): a systematic review of the literature with a new case report. J Otolaryngol Head Neck Surg.

[7] Alrawi M, McDermott M, Orr D, Russell J (2003). Nasal chondromesynchymal hamartoma presenting in an adolescent. Int J Pediatr Otorhinolaryngol.

[8] Li Y, Yang Q, Tian X, Li B, Li Z (2013). Malignant transformation of nasal chondromesenchymal hamartoma in adult: a case report and review of the literature. Histol Histopathol.

[9] Nakagawa T, Sakamoto T, Ito J (2018). Nasal chondromesenchymal hamartoma in an adolescent. Int J Pediatr Otorhinolaryngol Extra.

[10] Kim JE, Kim HJ, Ji HK, Ko YH, Chung SK (2009). Nasal chondromesenchymal hamartoma: CT and MR imaging findings. Korean J Radiol.

[11] Behery RE, Bedrnicek J, Lazenby A (2012). Translocation T(12;17)(Q24.1;Q21) as the sole anomaly in a nasal chondromesenchymal hamartoma arising in a patient with pleuropulmonary blastoma. Pediatr Dev Radiol.

[12] Chandra M, Sharma N, Venkatahalam VP (2014). Nasal chondromesenchymal hamartoma: a case report and review of literature. JK Pract.

